# Hematite Thin Films with Various Nanoscopic Morphologies Through Control of Self-Assembly Structures

**DOI:** 10.1186/s11671-015-0936-x

**Published:** 2015-05-23

**Authors:** Jingling Liu, Yong-Tae Kim, Young-Uk Kwon

**Affiliations:** SKKU Advanced Institute of Nanotechnology (SAINT), Sungkyunkwan University, Suwon, 440-746 Korea; Department of Chemistry, BK-21 School of Chemical Materials Sciences, Sungkyunkwan University, Suwon, 440-746 Korea

**Keywords:** Hematite, F127, Self-assembly, Spin coating

## Abstract

Hematite (α-Fe_2_O_3_) thin films with various nanostructures were synthesized through self-assembly between iron oxide hydroxide particles, generated by hydrolysis and condensation of Fe(NO_3_)_3_ · 6H_2_O, and a Pluronic triblock copolymer (F127, (EO)_106_(PO)_70_(EO)_106_, EO = ethylene oxide, PO = propylene oxide), followed by calcination. The self-assembly structure can be tuned by introducing water in a controlled manner through the control of the humidity level in the surrounding of the as-cast films during aging stage. For the given Fe(NO_3_)_3_ · 6H_2_O:F127 ratio, there appear to be three different thermodynamically stable self-assembly structures depending on the water content in the film material, which correspond to mesoporous, spherical micellar, and rod-like micellar structures after removal of F127. Coupled with the thermodynamic driving forces, the kinetics of the irreversible reactions of coalescence of iron oxide hydroxide particles into larger ones induce diverse nanostructures of the resultant films. The length scale of so-obtained nanostructures ranges from 6 nm to a few hundred nanometers. In addition to water content, the effects of other experimental parameters such as aging temperature, spin rate during spin coating, type of substrate, and type of iron reagent were investigated.

**Electronic supplementary material:**

The online version of this article (doi:10.1186/s11671-015-0936-x) contains supplementary material, which is available to authorized users.

## Background

Owing to the growth of nanotechnology by leaps and bounds in the present era, nanofabrication methods with facile and cost-effective technologies are particularly important [1–4]. Self-assembly has captured significant interest in meeting societal and economic goals not only for its efficient, clean, and low-cost processes but also for its facile design of impressive morphologies, thanks to that soft matters can derive various nanostructures from their inherent capacity of forming various phases, such as spherical micelles, cylindrical or rod-like aggregates, or vesicles by steering the surface energetics [5–11].

In the process of forming nanostructures through self-assembly, the crystal growth of inorganic parts is achieved through two fundamental mechanisms, Oswald ripening and oriented attachment [12–15]. The Ostwald ripening is defined as the growth of larger particles at the cost of smaller ones according to the Gibbs-Thompson equation and Fick’s principle. The oriented attachment is described as the oriented aggregation and growth by self-organization of adjacent particles with a common crystallographic structure. These two mechanisms are influenced by experimental conditions, such as concentration, pH, temperature, and the presence of auxiliary materials. Therefore, by coupling the Ostwald ripening with oriented attachment mechanisms, various morphologies of materials such as nanowires, nanotubes, and hierarchical structures can be readily achieved [16–20].

However, such variability of nanoscopic morphology seems to be limited to bulk forms of materials. Thin film counterparts have been explored partly because of their apparent advantages for device applications. Self-assembly structures in thin films have been achieved through the evaporation-induced self-assembly (EISA) mechanism. However, although the underlying physics and chemistry governing the polymer orientation and crystal growth must be essentially the same, it is still a big challenge to synthesize different morphologies by the EISA process. This is mainly because the fast oriented attachment mechanism is dominant in EISA. The rapid evaporation of solvent increases the difficulty in controlling the self-assembly direction, which prevents the further growth of inorganic species. Therefore, in order to fully exploit nanostructures for device applications through self-assembly, the problems of self-assembly adherent to the thin film forms need to be overcome.

Hematite (α-Fe_2_O_3_) is a very promising material for a wide range of applications, such as catalysis, gas sensors, medical applications, magnetic devices, energy storage and conversion devices, and so on, for its abundance, high stability, and environmental friendliness [21–24]. As is well known, the performance of a material is closely related to its nanoscopic morphology which, in turn, is governed by the synthetic method. Even though several impressive achievements in nanostructured hematite films of nanowires [21, 25, 26], nanobelts [27], nanotubes [28, 29], mesopores [30, 31], inverse opal [32], nanoflakes [33], and nanoparticles [34–37] have been obtained and utilized in photoelectrochemical water splitting, gas sensors and energy storage devices, the list is still limited compared with that of the bulk form [28–38]. Therefore, in order to comprehensively investigate and optimize the morphology-dependent performance, it is important to develop versatile synthesis methods.

Hematite films are generally synthesized by anodization of iron foil [27, 28], spin coating [31, 32], chemical vapor deposition [35], spray pyrolysis [36], electrodeposition [39], and hydrothermal method [40]. Among them, spin coating is the most versatile method because of its simplicity and adoptability. It is also capable of incorporating self-assembly which may be able to form diverse nanoscopic morphologies. However, to date, only mesoporous films or nanoparticles of hematite have been achieved by this approach. One of the reasons appears to be due to the difficulty in controlling the continuous growth of iron species. Therefore, methods to control the self-assembly process in spin coating may provide a breakthrough for the synthesis of diverse nanoscopic morphologies.

Herein, we explored humidity control as a way to sustain the growth environment of iron species by controlling the thermodynamics and kinetics of particle growth. The control of humidity can efficiently adjust the interfacial tension between water and inorganic species, which affects the nucleation, growth, and aging processes, leading to the control of the feature sizes and morphologies. The new strategy provides a promising potential for the controllable synthesis of various morphologies of metal oxides for various applications.

## Methods

### Syntheses of Hematite Films

The hematite films in this study were synthesized by spin coating a precursor solution on Si substrates, followed by aging under variously controlled environments and, then, by calcination [41, 42]. The precursor solution was prepared in the following steps: Fe(NO_3_)_3_ · 9H_2_O (1 g) was dissolved in anhydrous ethanol (5 ml), and the solution was stirred for 5 h at 40 °C. A Pluronic triblock copolymer F127 ((EO)_106_(PO)_70_(EO)_106_, EO = ethylene oxide, PO = propylene oxide) (400 mg) was added to this solution, and the solution was stirred for additional 2 h. This coating solution was spin-coated or drop-coated without spinning on Si substrates under a controlled humidity level of 60 % relative humidity (RH) at 24–26 °C. The coated films were aged under a controlled humidity level and at a designated temperature. For this purpose, homemade aging chambers were constructed and used (ESI Additional file 1: Figure S1). Inside the chamber, water is placed on the bottom and samples are suspended above the water level so that samples can be exposed to water vapor. The humidity level inside the chamber was controlled by using water saturated with different salts. According to the literature, aqueous solution saturated with LiCl, MgCl_2_, NaBr, and NaCl generate humidity levels of 10, 26, 51, and 75 % RH, respectively. In addition, 0 % RH condition was generated by having no water and 100 % RH condition by having pure water. The chamber was tightly sealed and placed in an oven whose temperature was set at 18, 40, 60, and 80 °C. After aging, the films were heated at 400 °C in air with the ramping rate of 1 °C/min for 2 h to remove organics and volatiles. In addition to the humidity level, we varied other process parameters. We varied the spinning rate to 0 (drop coating), 500, and 1000 rpm while 5000 rpm was used in most of cases. We investigated the effect of substrate by using quartz or fluorine-doped tin oxide (FTO) substrate while Si substrates were used in all of the cases unless specified. Finally, we tried using Fe(acac)_3_ and FeCl_3_ · 6H_2_O in the place of Fe(NO_3_)_3_ · 9H_2_O.

Hematite films in this study will be denoted as *FX* (temp, RH, spin rate), where *X* stands for the iron reagent used, *X* = N for Fe(NO_3_)_3_ · 9H_2_O, *X* = A for Fe(acac)_3_, and *X* = C for FeCl_3_ · 6H_2_O. A spin rate of 5000 rpm will not be indicated in this notation.

### Characterization

The morphologies were observed via field emission scanning electron microscopy (FE-SEM, JEOL JSM-7401 F) and field emission high-resolution transmission electron microscopy (HRTEM, JEM-2100 F). Chemical bonding and oxidation states were investigated by X-ray photoelectron spectroscopy (XPS, Perkin-Elmer PHI 660). Phase identity was made by Raman spectroscopy, which was recorded using a 514-nm (2.6 mW) line of Ar^+^ laser (Renishaw, Invia).

## Results and Discussion

In the present study, hematite thin films with various morphologies via the EISA process were successfully obtained by modulating experimental parameters including humidity, temperature, spin coating rate, and type of precursors. The synthetic conditions and their resultant morphologies are summarized in Table 1. It can be seen that the morphologies are strongly affected by the experimental conditions, specially the aging humidity. The effects of each of the experimental parameters on the morphology are discussed in detail in the following. We also investigated the effect of calcination temperature. However, temperature does not appear to induce morphological changes except that too high temperatures (~600 °C) partly collapse the morphology (Additional file 1: Figure S2). For this reason, the calcination temperature was fixed at 400 °C in this study.

**Table 1:** List of synthesis conditions of various nanoscopic hematite thin films

Precursor	Temperature (°C)	Spin rate (k/rpm)	Humidity (%)	Aging time (day)	Symbols	Morphologies
Fe(NO_3_)_3_ · 9H_2_O	18	5	75	2	FN(18 °C, 75 %)	Non-uniform film
	40	5	75	2	FN(40 °C, 75 %)	Bundle-like
	60	5	75	2	FN(60 °C, 75 %)	NW
	80	5	0	2	FN(80 °C, 0 %)	MP
	80	5	10	2	FN(80 °C, 10 %)	Distorted MP
	80	5	26	2	FN(80 °C, 26 %)	Urchin-like
	80	5	51	2	FN(80 °C, 51 %)	Distorted urchin-like
	80	5	75	2	FN(80 °C, 75 %)	NW
	80	5	100	2	FN(80 °C, 100 %)	Bundle-like
	80	0	75	2	FN(80 °C, 100 %, 0 k)	Bundle-like
FeCl_3_ · 6H_2_O	80	5	75	2	FN(80 °C, 75 %)	Flower-like
Fe(acac)_3_	80	5	75	2	FN(80 °C, 75 %)	Non-uniform film

*NW* network nanowires, *MP* mesoporous, *0 k* 0 rpm, *5 k* 5000 rpm

One can expect that they are hematite unless there is a kinetic trap during the syntheses. As a support for this, we prepared a bulk sample by using the same precursor solution aged at 80 °C for 2 days followed by calcination. Its X-ray diffraction (XRD) pattern (shown in Additional file 1: Figure S3) matches well with the hematite in the literature (JCPDS No.: 89–0597). Unfortunately, XRD patterns of the thin film samples could not be obtained because of the extremely small quantities of materials in the samples. On the other hand, all of the analyses data including HRTEM along with fast Fourier transform (FFT) patterns, XPS (Additional file 1: Figure S4), and Raman spectroscopy (Additional file 1: Figure S5) data all indicate that all of the samples are hematite.

### Effects of Aging Humidity

**Fig. 1:**
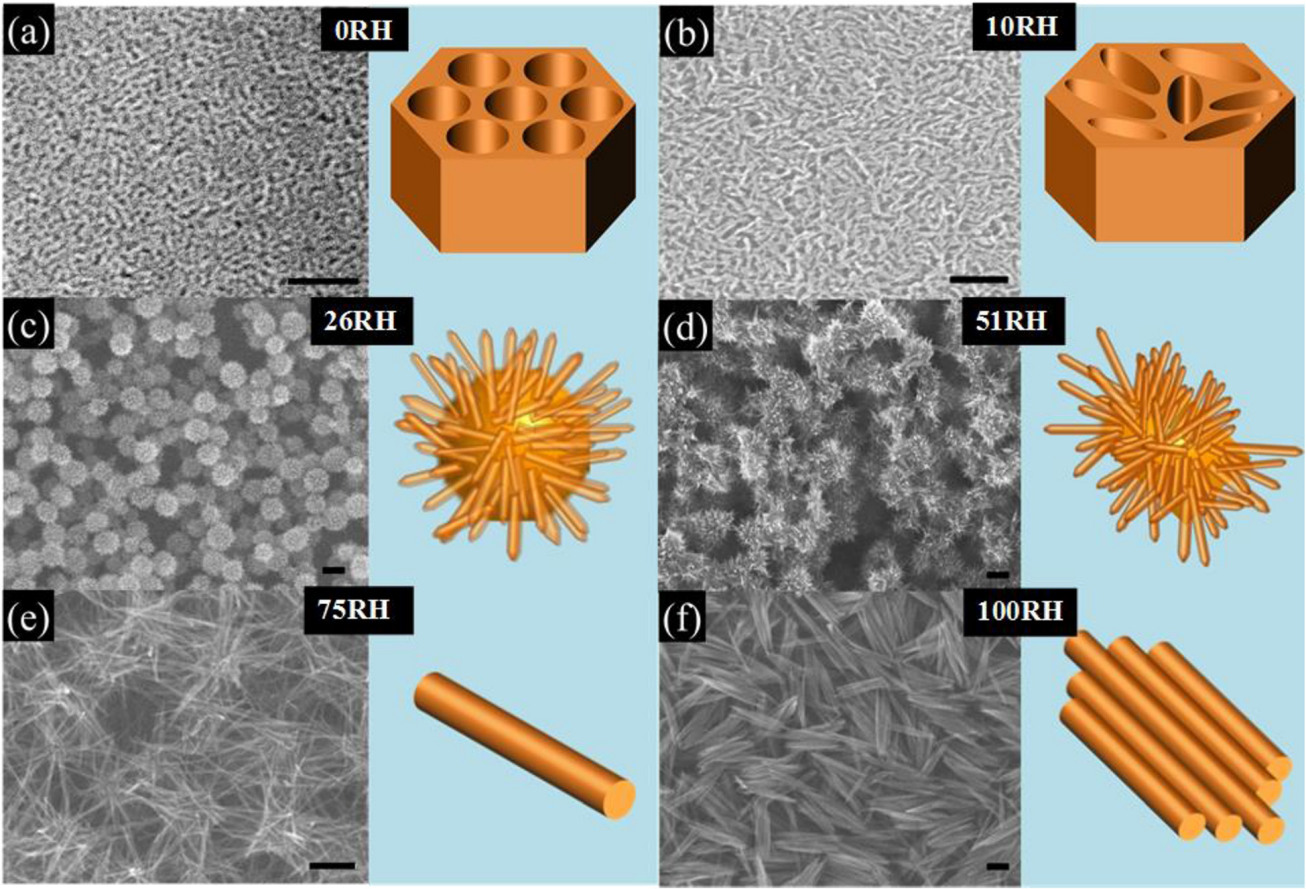
SEM images and schematic drawing of hematite thin films at different aging humidity levels. **a** FN(80 °C, 0 %); **b** FN(80 °C, 10 %); **c** FN(80 °C, 26 %); **d** FN(80 °C, 51 %); **e** FN(80 °C, 75 %); and **f** FN(80 °C, 100 %). The *scale bar* 100 nm

**Fig. 2:**
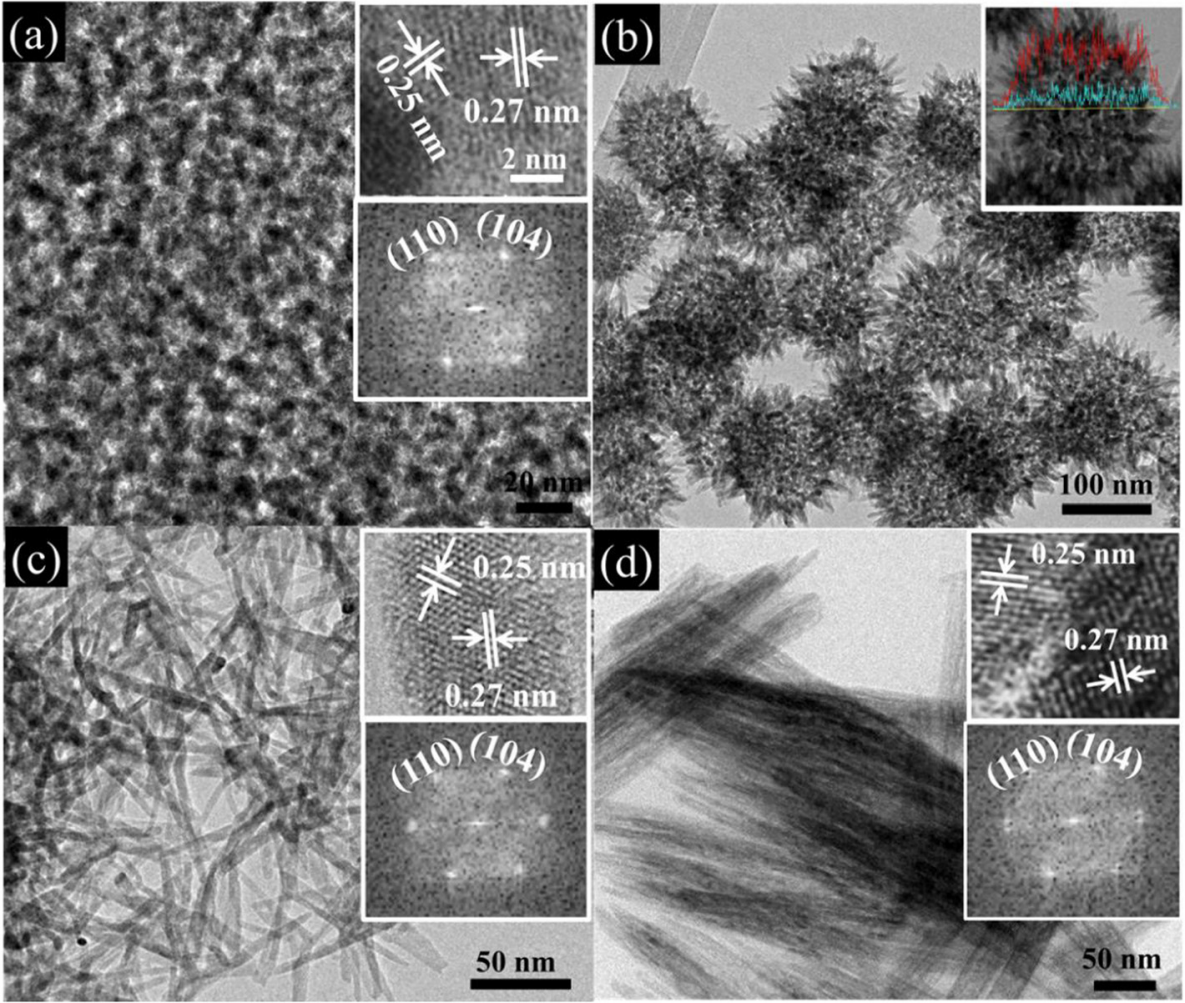
TEM images of hematite thin films at different aging humidity levels. **a** FN(80 °C, 0 %); **b** FN(80 °C, 26 %); **c** FN(80 °C, 75 %); and **d** FN(80 °C, 100 %). **a**, **c**, **d** The *upper insets* are high-resolution TEM images and the *lower insets* the corresponding FFT. **b** The *inset* shows the EDX line profiles of Fe (*red*) and O (*blue*)

The various morphologies obtained with different aging humidity levels are shown in Figs. 1 and 2. Other parameters were kept constant: Fe(NO_3_)_3_ · 9H_2_O was used, the aging temperature was 80 °C, and the spin rate was 5 k. At 0 % RH of aging humidity, a mesoporous structure with ~9-nm-sized pores and ~5-nm-thick walls was formed (Figs. 1a and 2a). Although the pore ordering is irregular, indicating a wormlike pore structure, the pore size is uniform throughout the film. As the aging humidity increases to 10 % RH (Fig. 1b), the pores become more irregular, but the average pore size is almost unchanged from that of 0 % RH. At 26 % RH, a hierarchical structure was formed, consisting of ~120-nm spheres with protruding thorns with ~10 nm in diameter and ~40 nm in length (Figs. 1c and 2b). The EDX line profiles for Fe and O elements are almost flat across the sphere, even with a small drop in the center, indicating a hollow sphere (inset of Fig. 2b). When the aging humidity reaches 51 % (Fig. 1d), the hollow spheres become irregular and the thorns grow longer. When the aging humidity reaches 75 %, the spheres disappear and nanowires with a uniform diameter of ~6 nm form. These nanowires form a network structure with pores between nanowires (Figs. 1e and 2c). When the aging humidity reaches 100 %, nanowire bundles formed. The nanowire bundles are ~500 nm in length. The nanowires in the bundle have a uniform thickness of ~50 nm.

For most of the samples, we took HRTEM images. The HRTEM images (upper insets of Fig. 2a, c, d) show lattice fringes from which the (104) and (110) planes of hematite with the *d* values of 0.27 and 0.25 nm, respectively, can be identified. The indexing of the lattice fringes was confirmed by the corresponding FFT patterns (lower insets of Fig. 2a, c, d). The ED pattern taken on the FN(80 °C, 75 %) sample is identical to these FFT patterns. These FFT patterns agree well with the SAED pattern taken on the FN(80 °C, 75 %) sample (ESI Additional file 1: Figure S6).

### Effects of Aging Temperature

**Fig. 3:**
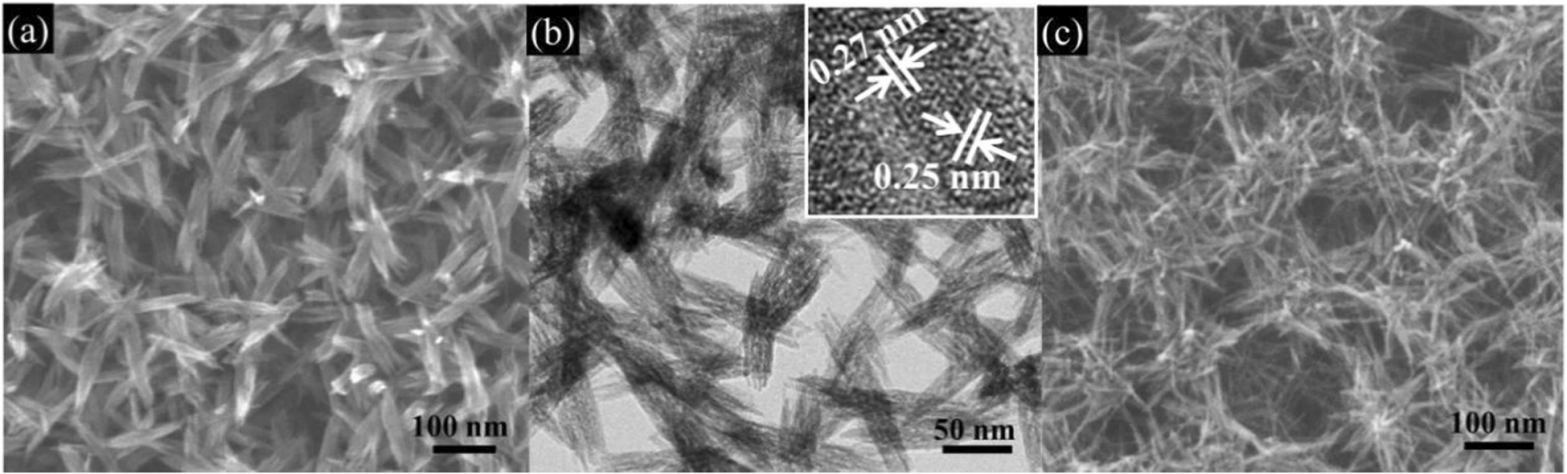
SEM images of hematite thin films at different aging temperatures. **a** FN(40 °C, 75 %); **b** the TEM image of FN(40 °C, 75 %), and **c** FN(60 °C, 75 %). **b** *Inset* is the HRTEM image of FN(40 °C, 75 %)

The aging temperature is another important parameter to induce the morphological evolution of hematite films. Different temperatures of 18, 40, 60, and 80 °C were investigated at 75 % RH. The differences in morphology at different aging temperatures can be seen from Fig. 3. At a low temperature of 18 °C, only a non-uniform porous-like film was observed (ESI Additional file 1: Figure S7). As the temperature increases to 40 °C, nanowire bundles with ~100–200 nm in length and ~30 nm in diameter are formed as presented in Fig. 3a, b. Lattice fringes of the (104) and (110) planes of hematite with *d* values of 0.27 and 0.25 nm, respectively, can be identified in the HRTEM image (inset of Fig. 3b). When the aging temperature reaches 60 °C (Fig. 3c), a network structure of nanowires formed, which is similar to that obtained at 80 °C (Fig. 1e). However, the FN(80 °C, 75 %) sample (Fig. 1e) is more uniform in nanowire diameter and longer in length of nanowires compared with the FN(60 °C, 75 %) sample.

### Effect of Spinning Rate

**Fig. 4:**
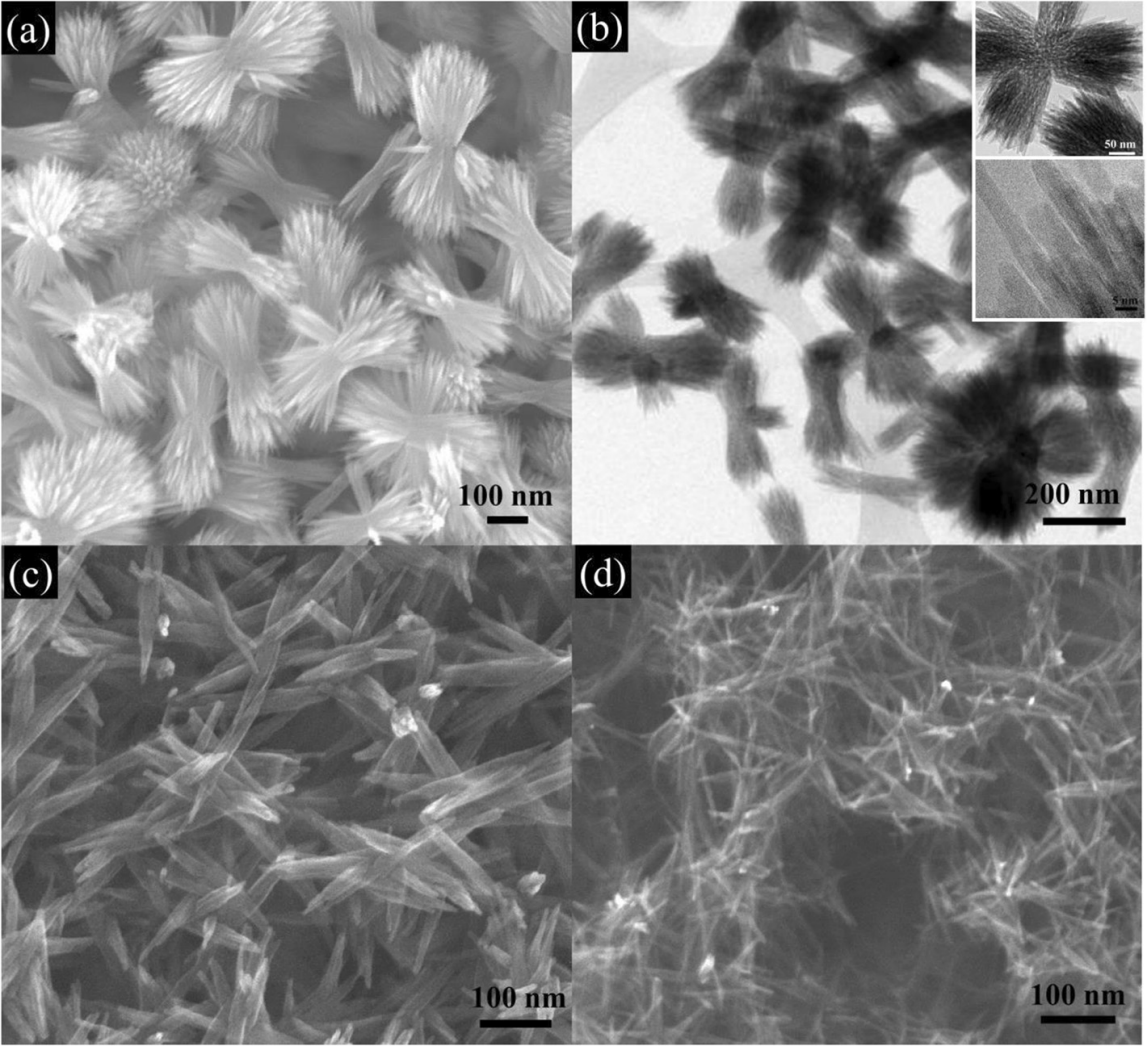
Spin rate dependence of morphologies of hematite thin films aged for 2 days. **a** FN(80 °C, 75 %, 0 k); **b** TEM image of the FN(80 °C, 75 %, 0 k) sample; **c** FN(80 °C, 75 %, 0.5 k), and **d** FN(80 °C, 75 %, 1 k)

Figure 4 shows the images of hematite films obtained by drop coating and spin coating with low spin rates. The samples were aged at 80 °C under 75 % RH. As seen in Fig. 4a, b, straw bundle-like architectures formed when the film was prepared by drop coating. Each straw bundle possesses two fantails consisting of bundles of outward-spreading nanorods that are bonded to each other in the center. The nanowire in the bundle is ~300 nm long and ~6 nm thick. As the spin rate is increased to 500 rpm (Fig. 4c) and to 1000 rpm (Fig. 4d), the morphology is gradually transformed into bundles of nanowires and to a nanowire network. The latter morphology is similar to that from the 5000-rpm spin rate condition (FN(80 °C, 75 %)).

### Effect of Substrate

The effect of substrate on the morphology was investigated by using quartz and FTO in place of a Si wafer for the synthesis of FN(80 °C, 75 %) (ESI Additional file 1: Figure S8). The morphologies of these films are practically the same as those of the Si substrate in Fig. 1e. This means that, at least for the case of FN(80 °C, 75 %) and probably for others as well, the nature of the substrate does not influence the morphology. By this, one can rule out the possibility of heterogeneous nucleation at the substrate-film material interface in the formation mechanism of the nanostructures of hematite films. The insensitivity to substrate of morphology may be of importance if one tries to make use of the nanostructured hematite films in device applications.

### Effect of Iron Reagent

The choice of a right reagent for the iron source is important for the successful generation of diverse nanostructures. We have tried Fe(acac)_3_ or FeCl_3_ · 6H_2_O instead of Fe(NO_3_)_3_ · 9H_2_O for the synthesis of FN(80 °C, 75 %). The results (ESI Additional file 1: Figure S9) are completely different from that of FN(80 °C, 75 %). The reaction with Fe(acac)_3_ produced an inhomogeneous film. Fe(acac)_3_ has a strong metal-ligand binding, mainly due to the chelate effect of the bidentate ligand acac, and does not allow the generation of iron oxide hydroxide species, an indispensible component for the self-assembly structures. The reaction with FeCl_3_ · 6H_2_O also produced an inhomogeneous flower-like nanostructure with ~300-nm needles. Cl^−^ ions are well known to participate in the crystallization of β-FeOOH. Therefore, the use of FeCl_3_ · 6H_2_O strongly facilitates the growth of needle-like β-FeOOH crystals that are too large to take part in the self-assembly structures [43].

**Fig. 5:**
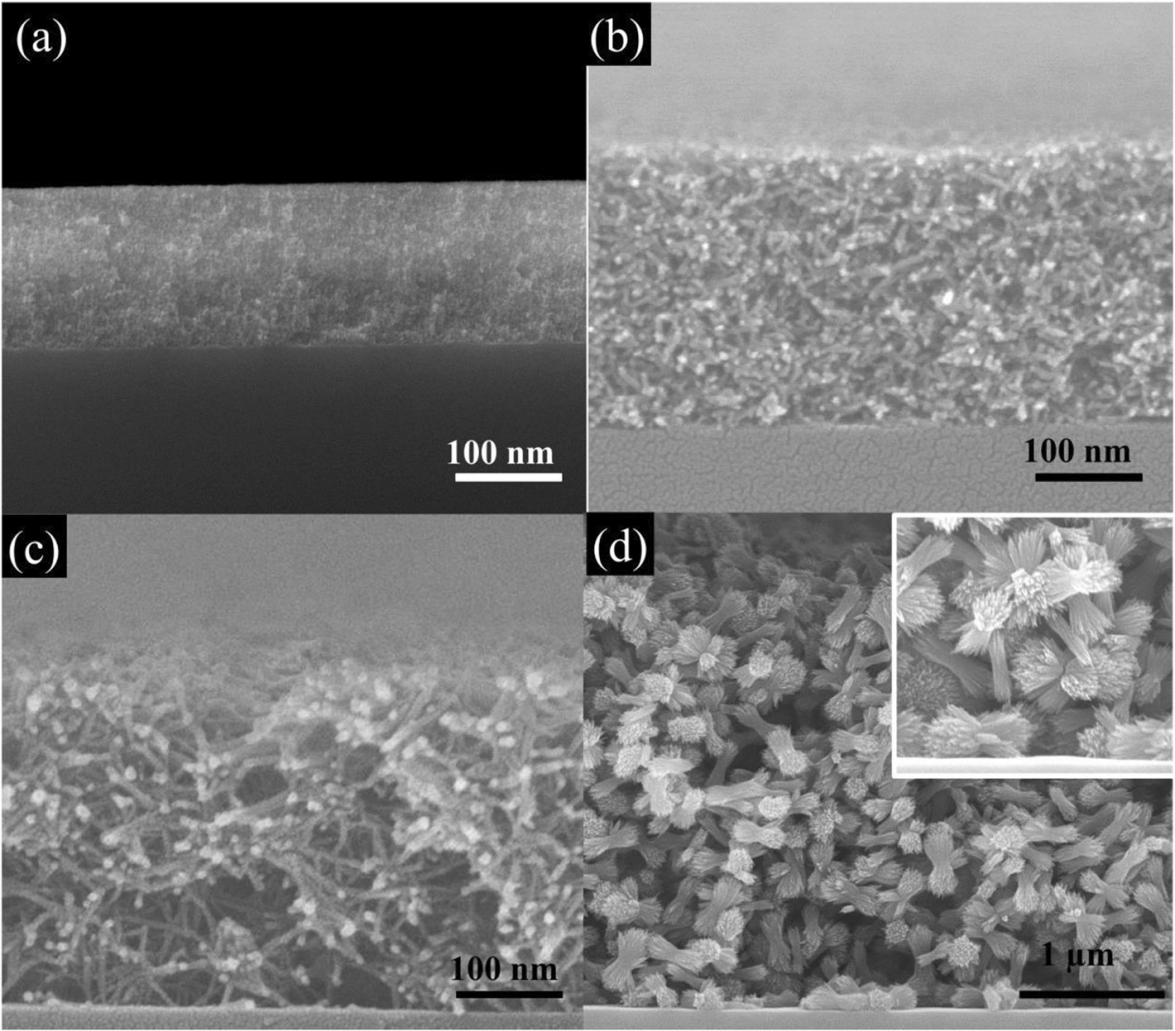
Cross section images of various morphologies of hematite thin film. **a** FN(80 °C, 0 %, 5 k) aged for 2 days; **b** FN(80 °C, 75 %, 5 k) aged for 10 h, **c** FN(80 °C, 75 %, 5 k) aged for 2 days; and **d** FN(80 °C, 75 %, 0 k) aged for 2 days

Our thin film samples are uniform as can be seen in the cross section images in Fig. 5. FN(80 °C, 0 %), FN(80 °C, 75 %), and FN(80 °C, 0 %, 0 k) have different thicknesses of ~175 nm, ~315 nm, and ~2.8 μm, respectively, and the thicknesses are uniform across the films (Fig. 5a, c, d). The cross section image of FN(80 °C, 75 %) aged for 10 h is also presented in Fig. 5b. It is ~270 nm in thickness. Compared with FN(80 °C, 75 %) aged for 2 days, it is thinner and the constituting nanowires are shorter and the network is not well developed. However, the film is uniform in the whole area, suggesting that the evolution of morphology is taking place uniformly throughout the film.

### Mechanism of Nanostructure Formation

The various morphologies obtained in the present study can be explained in terms of thermodynamic driving forces to attain the most stable self-assembly structures under given conditions and often opposing kinetic constrains due to the irreversible condensation reaction of iron species and the mobility of the film materials. Both the thermodynamic and kinetic factors are functions of composition including water content in the films, which can be controlled by the humidity level in the surrounding. The possibility of heterogeneous nucleation by substrates affecting the nanostructures can be ruled out to see that the same nanostructures are obtained regardless of the type of substrate.

All of the nanostructures of the present study have started from the identically prepared as-cast films. Since the precursor solution has been stirred in air for 5 h, it is likely that the Fe^3+^ ions are condensed into iron oxide hydroxide particles, probably sub-nanometer in size. Therefore, the as-cast films are mainly composed of iron oxide hydroxide particles and F127. Most of the solvent ethanol would have been lost by evaporation before the as-cast films were placed in the aging chamber. However, there must be some ethanol left in the film, at least, for a while so that the film materials (iron oxide hydroxide particles and F127) can move around to attain a self-assembly structure. From the morphology of the FN(80 °C, 0 %) sample, which has not experienced the effect of water and thus is expected to maintain the structure of the as-cast film, we can infer that the self-assembly structure of the as-cast film is composed of rod-like micelles whose inside is filled with removable organics and outside is occupied by iron oxide hydroxide particles. Therefore, in the as-cast film state, the hydrophobic PO blocks of F127 are segregated into micelle rods and the hydrophilic EO blocks are associated with the iron oxide hydroxide particles forming hydrophilic domains.

Depending on the humidity level in the aging environment, different amounts of water molecules are introduced to the film material. The water content in the film material affects the final morphology in two ways. Thermodynamically, water content directs the most stable self-assembly structure through the change of the volume ratio between the hydrophilic and hydrophobic domains. Kinetically, when water content is sufficient, water molecules can function as a mobilization medium of the film materials so that thermodynamically stable structures can be attained. Conversely, when the water content is lower than a mobilization threshold, the most stable self-assembly structure cannot be reached and the final structure is a result of kinetic trapping. Water content also influences the rate of condensation between small iron oxide hydroxide particles into larger particles. If the water content is high, the condensation reaction is suppressed and the mobility within the film material may remain high enough to allow the thermodynamic structure to be formed. On the contrary, if the water content is low, iron oxide hydroxide particles are given more chances to condense into larger particles, the film material will become progressively rigid, and the final morphology will reflect the time progression of the interplay between thermodynamics and kinetics.

**Fig. 6:**
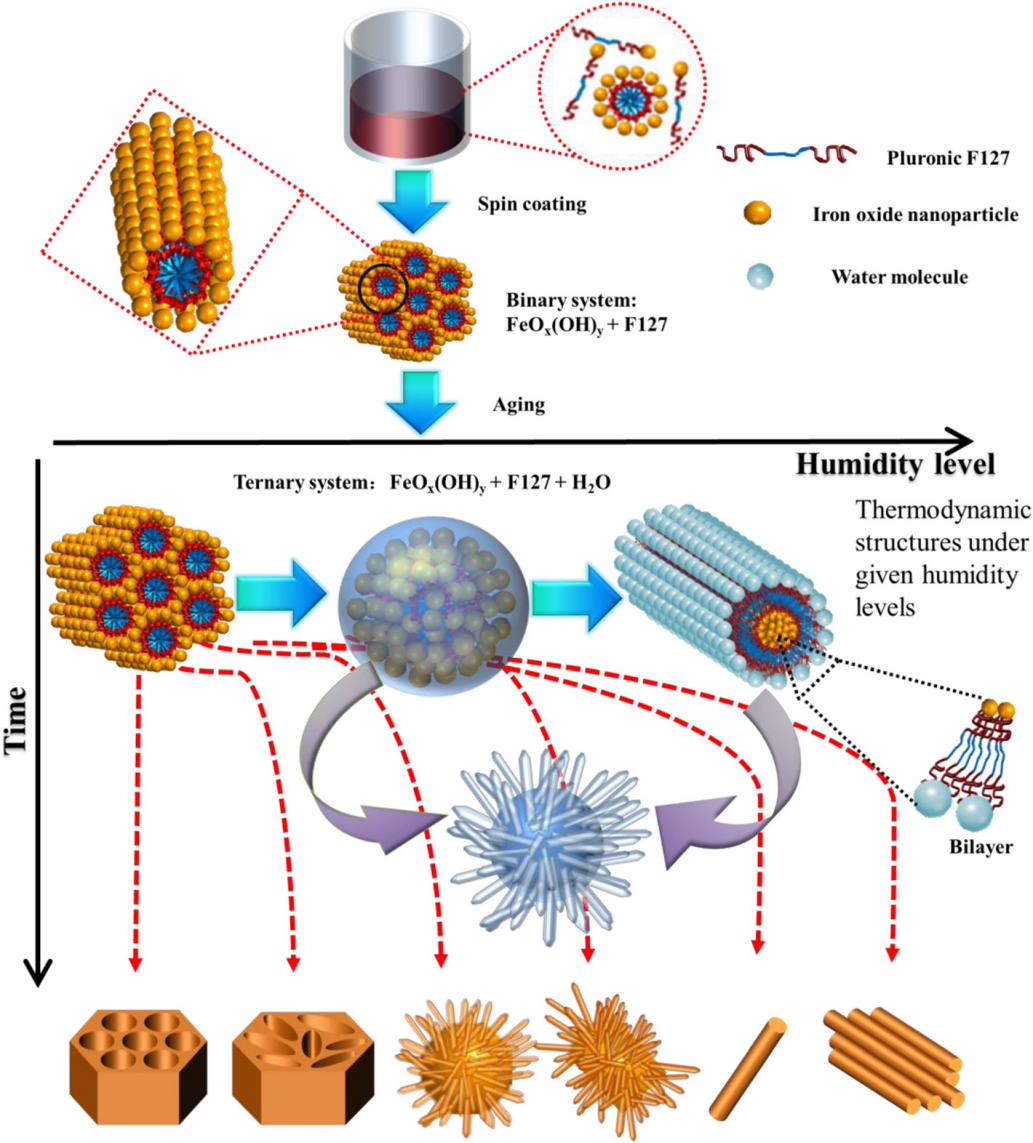
Proposed mechanism of various humidity-dependent morphologies of hematite thin films

Figure 6 summarizes these ideas and shows the pathway for each of the nanostructures obtained in the present study. From the results, we consider that there are three different thermodynamically stable structures. At low water content, the volume ratio between the hydrophilic and the hydrophobic domains is low and the most stable structure is the hexagonal structure with the PO hydrophobic domain segregated into ~10-nm-sized rod-like micelles. At high water content, F127 molecules form a bilayer-type structure with one side associated with iron oxide hydroxide species and the other side with water. Since the volume of water is larger than that of iron oxide hydroxide species, the latter forms a rod-like morphology. Between these two extremes, the F127 bilayer forms spherical micelles. The iron oxide hydroxide species are accumulated inside the spheres and eventually form a hematite shell on the internal surface of the micelle. A similar phase behavior depending on the volume ratio has been observed in a triblock poly(ethylene oxide)-*block*-poly(allyl glycidyl ether)-*block*-poly(*tert*-butyl glycidyl ether) copolymer system [44].

In addition to the thermodynamically stable structures, one has to consider the kinetic effect when the water content is not high. In the case of the 0 % RH condition, however, the kinetic factor does not influence the structure because the stable structure is already achieved in the as-cast film stage. On increasing the humidity level to 10 % RH, the stable structure is shifted to the micellar one, but the strongly restricted kinetics does not allow full movement of film materials and the final film shows morphology which is a small distortion from the one from the 0 % RH condition. On further increasing the water content to 26 % RH, the film material becomes mobile and the thermodynamically stable micelle structure is obtained. The hematite spheres have short protrusions which may be because the composition is slightly high in water content than the optimal composition for the pure micelle phase so that the rod-like morphology is formed on the surface of the micelles. This behavior is continued when the humidity level is increased to 51 % RH whose product is composed of coalesced micelles and longer nanowires. It is also possible that these two urchin-like nanostructures are the results of the combination of the abovementioned thermodynamics and kinetics, that is, the micelles are formed as a thermodynamic product and the nanowire protrusions are formed in the later stage as a result of shifted composition after the formation of the micelles. At 75 % RH, all of the iron species has been used to form nanowires probably because the high degree of mobility allows the full attainment of the thermodynamic structure. Upon further increase of humidity level to 100 % RH, some of the F127 molecules are dissolved in water and unavailable for the self-assembly. The net effect is to lower the content of F127, and one way for the system to react to such a change is to aggregate the existing nanowires into bundles.

The two additional morphologies obtained by using a lower spinning rate can be explained with this scheme. When the solution is drop-cast, the film material is much thicker than in the spin coating cases. Evaporation of ethanol is not complete and ethanol is included in the self-assembly structure. Because ethanol is relatively hydrophobic, it is likely to be associated with the PO block. If the self-assembly structure is micelle, the shell thickness is increased because of the increased volume of the PO part by the inclusion of ethanol. This will reduce the curvature of the micelle, increasing the micelle size. The iron oxide hydroxide particles are not well condensed because the major solvent is ethanol. But ethanol is replaced by water during the aging, and the micelle will be collapsed because of the incomplete condensation. As the water content increases, nanowires will grow on the surface of existing collapsed micelles resulting in the straw bundle-like morphology. As the spin rate is increased to 500 rpm, the evaporation of ethanol is increased but the as-cast film still contains a large amount of ethanol. The reduced ethanol content makes the micelle size smaller than in the drop-casting case. The remaining mechanism is similar as in the drop-casting case, the net result of which is the straw bundle-like morphology with reduced sizes in the short dimension but increased sizes in the longer dimension.

In the lower part of Fig. 6, we plot the obtained nanostructures as a function of humidity level and temperature. In this plot, temperature affects the widths of stability fields but does not induce any dramatic effects on the stable phases. The major role of temperature is, therefore, to facilitate the attainment of the final self-assembly structures.

## Conclusions

In this study, a novel, simple EISA-based method was developed for synthesizing various nanostructures of hematite thin films. We devised a system in which the water content critically determines the stable self-assembly structures. Through controlling the relative humidity in the surrounding, we could tune the thermodynamic driving forces. The thermodynamics coupled with the kinetics of the irreversible reactions in the film material direct the final nanoscopic morphologies. As a result, we obtained various nanostructured hematite thin films ranging from mesoporous, through urchin-like, to nanowire network, and to nanowire bundle structures. The length scale of these nanostructures ranges from a few nanometers to a few micrometers, thanks to the wide range of self-assembly structures available. Based on the obtained morphologies, a formation mechanism is proposed. Synthesis parameters such as temperature of aging, spin rate in the spin coating, type of substrate, and type of iron reagent were explored, and the results agree with the global picture of the proposed mechanism. We believe that our findings can be applied to other materials than hematite and, thus, can lead to various nanostructured thin films of them, which will be the first step in optimizing material design for their specific applications.

## Additional file

Additional file 1: **Electronic Supplementary Information (ESI) available: Hematite thin films with various nanoscopic morphologies through control of self-assembly structures.**
