# Supplementary material for: Hematite Thin Films with Various Nanoscopic Morphologies Through Control of Self-Assembly Structures
Source: Nanoscale Res Lett. 2015 May 23;10:228. doi: 10.1186/s11671-015-0936-x (PMC4444645; doi:10.1186/s11671-015-0936-x)
Supplement: Supplementary file 1 — Electronic Supplementary Information (ESI) available: Hematite thin films with various nanoscopic morphologies through control of self-assembly structures. [file 11671_2015_936_MOESM1_ESM.docx]

**Hematite Thin Films with Various Nanoscopic Morphologies through Control of Self-Assembly Structures**

Jingling Liu^1^
Email: jingling@skku.edu

Yong-Tae Kim^1^

Email: tanatos@skku.edu

Young-Uk Kwon^1, 2,*^

*Corresponding author

Email: ywkwon@skku.edu

^1^SKKU Advanced Institute of Nanotechnology (SAINT), Sungkyunkwan University, Suwon 440-746, Korea

^2^Department of Chemistry, BK-21 School of Chemical Materials Sciences, Sungkyunkwan University, Suwon 440-746, Korea


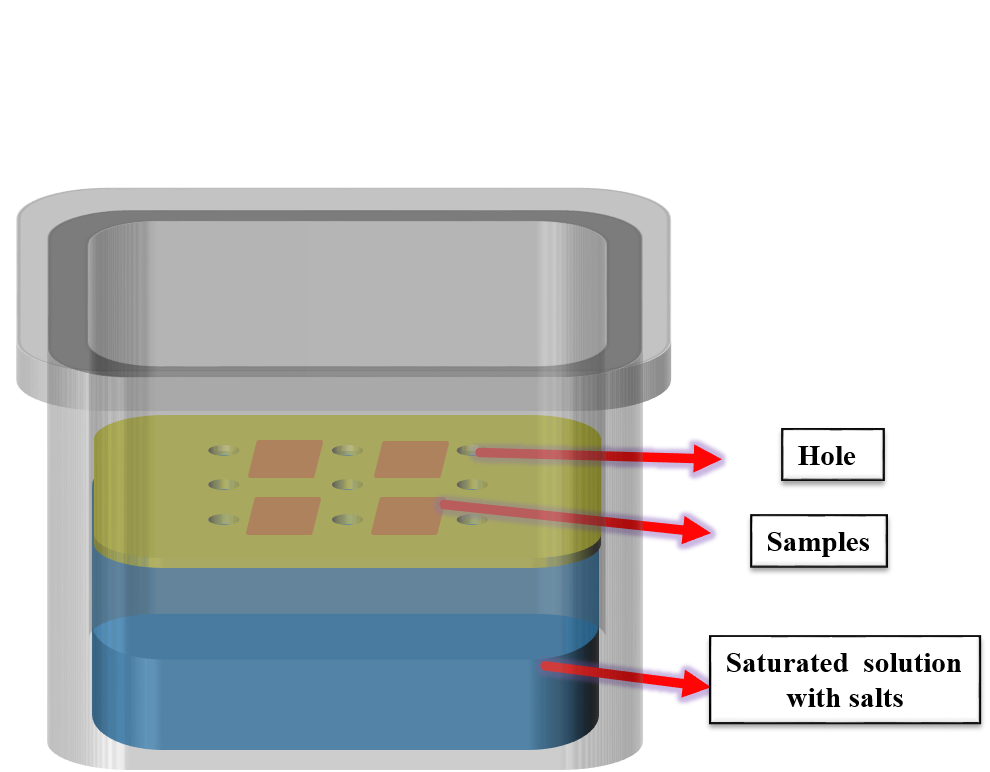


**Figure S1** Schematic of a homemade chamber used for the aging of hematite films under controlled humidity levels.


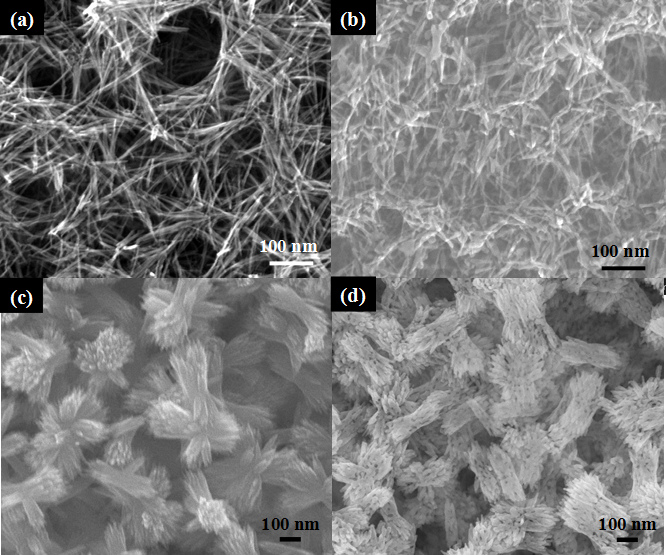


**Figure S2** SEM images of selected samples calcined at different temperatures: (a) FN(80°C, 75%) at 500 °C, (b) FN(80°C, 75%) at 600 °C, (c) FN(80°C, 75%, 0k) at 500 °C and (d) FN(80°C, 75%, 0k) at 600 °C.


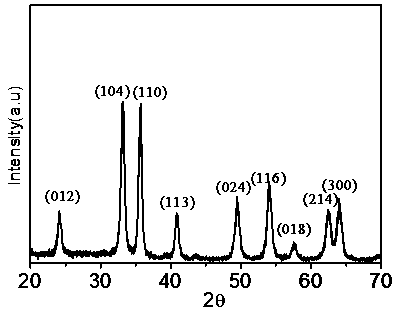


**Figure S3** XRD patterns of the bulk form prepared by the same precursor solution aged at 80 °C for 2days without the control of humidity (JCPDS NO. 89-0597).


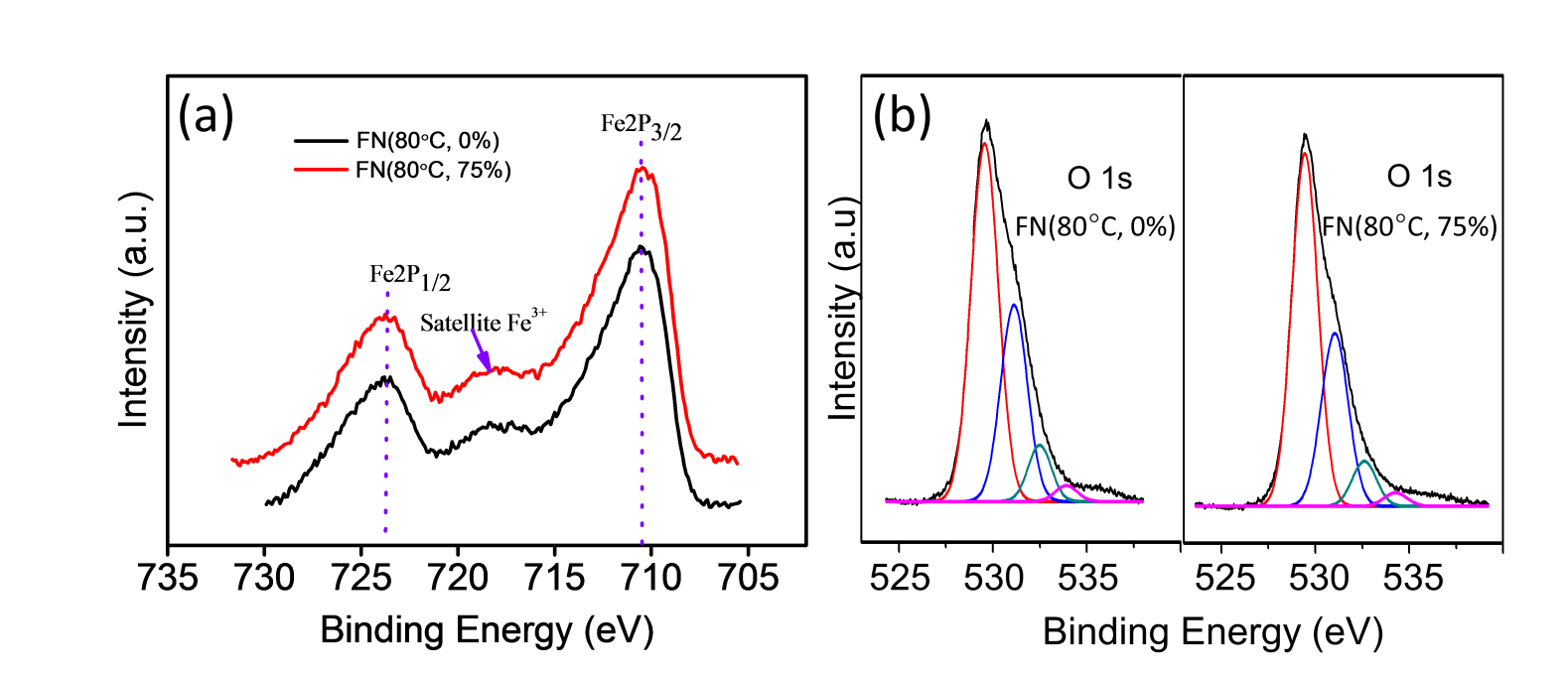


**Figure S4** XPS spectra of (a) Fe 2p, and (b) O 1s for FN(80°C, 0%) sample and FN(80°C, 75%) samples.


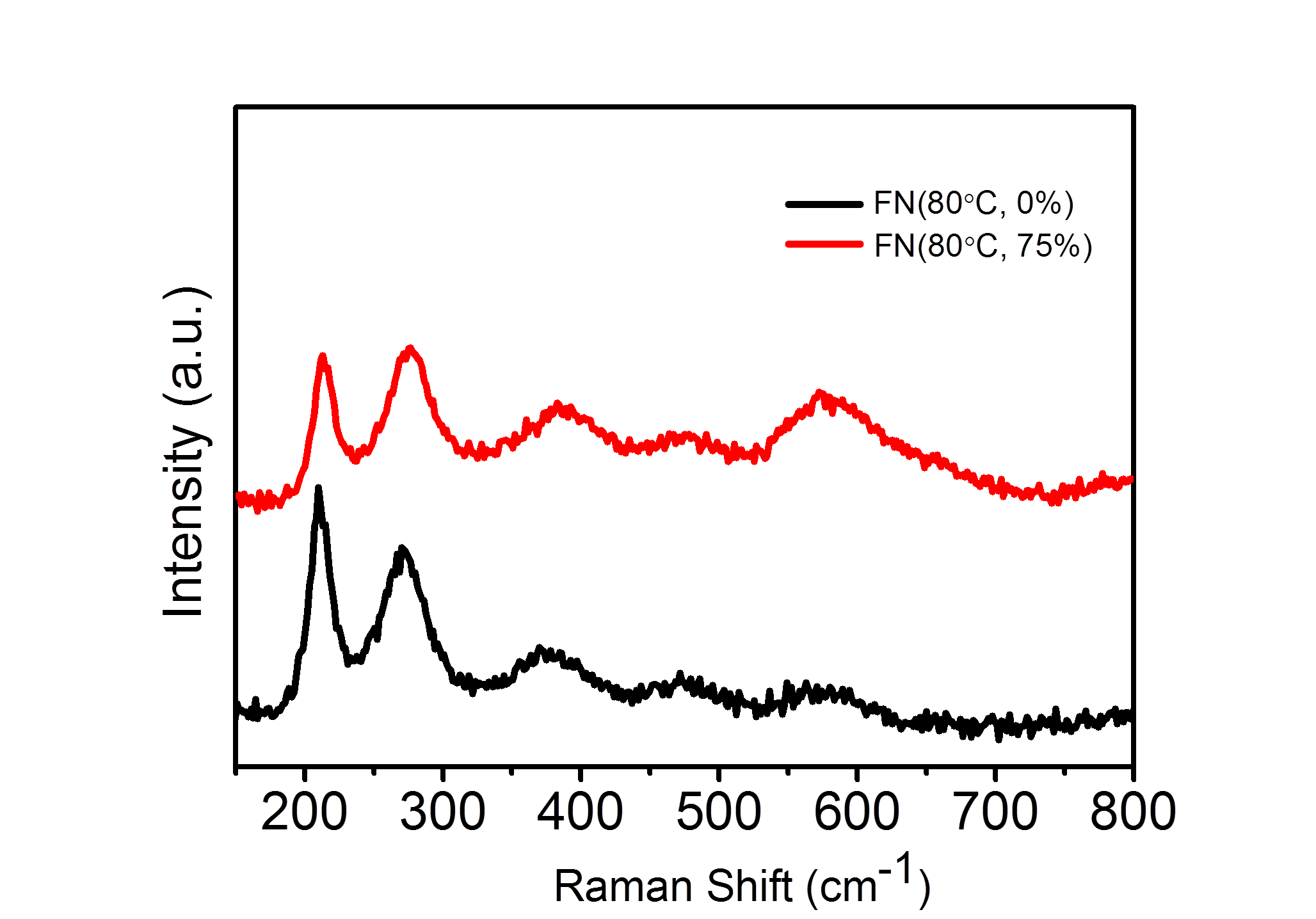


**Figure S5** Raman spectra of FN(80°C, 0%) sample and FN(80°C, 75%)samples.


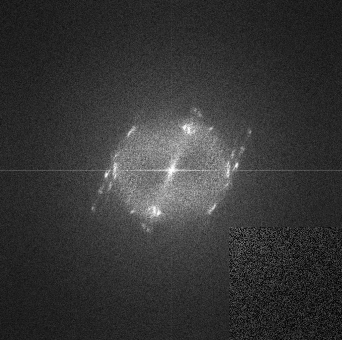


**Figure S6** SEAD pattern of FN(80°C, 75%) sample. The film was prepared with the spin rate of 5k rpm and aged for 2 days.


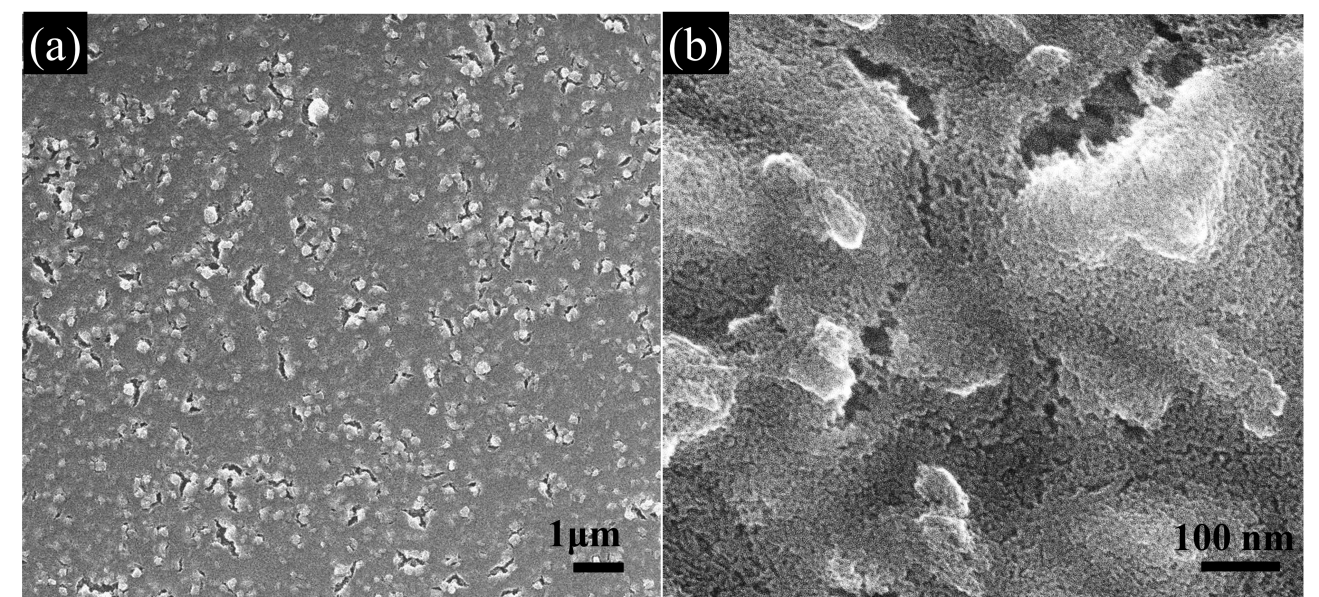


**Figure S7** SEM images of FN(18°C, 75%) sample at different magnifications. The film was prepared with the spin rate of 5k rpm and aged for 2 days.


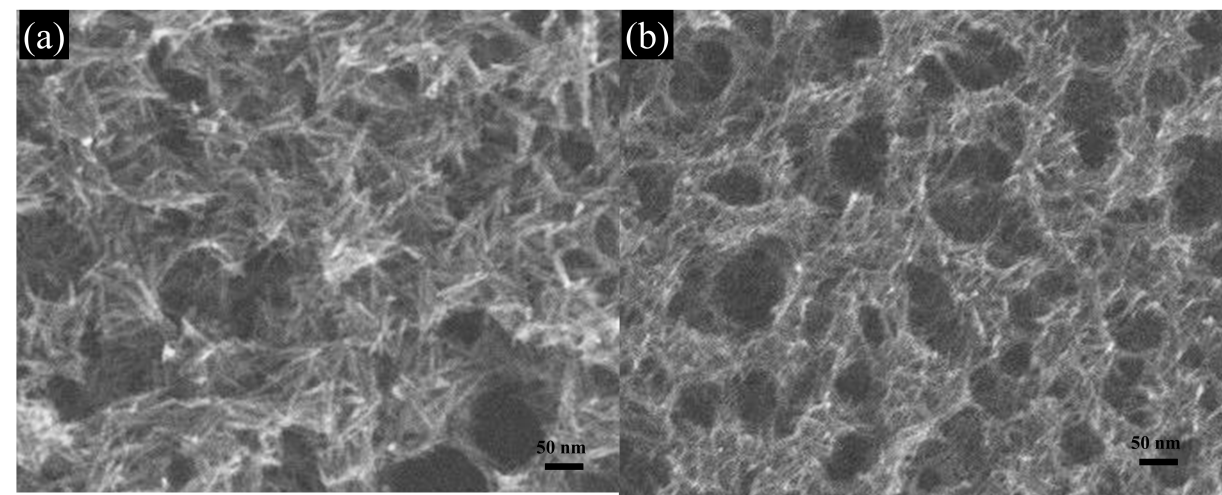


**Figure S8** The substrate dependent morphologies of hematite thin films. (a): FN(80°C, 75%) sample on quartz substrate, (b): FN(80°C, 75%) sample on FTO substrate.


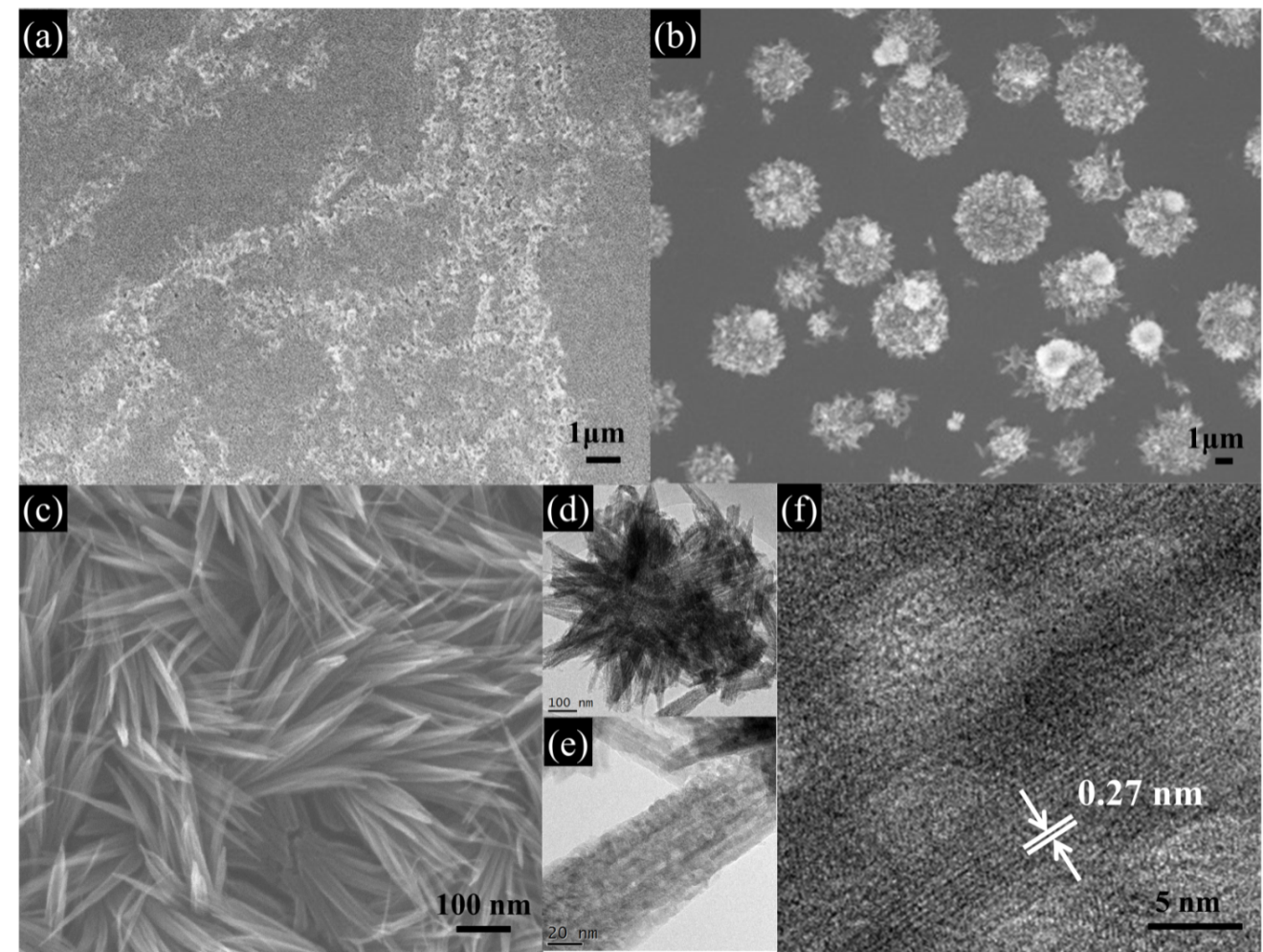


**Figure S9** The images of hematite thin films using different iron precursors. (a) FA(80°C, 75%), and (b and c) FC(80°C, 75%), and (d, e and f) are TEM images of FC(80°C, 75%) sample.
